# Pollen production of downy birch (*Betula pubescens* Ehrh.) along an altitudinal gradient in the European Alps

**DOI:** 10.1007/s00484-023-02483-7

**Published:** 2023-05-08

**Authors:** Surendra Ranpal, Susanne von Bargen, Stefanie Gilles, Daria Luschkova, Maria Landgraf, Claudia Traidl-Hoffmann, Carmen Büttner, Athanasios Damialis, Susanne Jochner-Oette

**Affiliations:** 1 Physical Geography/Landscape Ecology and Sustainable Ecosystem Development, Catholic University of Eichstätt-Ingolstadt, 85072 Eichstätt, Germany; 2 Albrecht Daniel Thaer-Institute for Crop and Animal Sciences, Division Phytomedicine, Humboldt-University of Berlin, Berlin, Germany; 3 Environmental Medicine, Faculty of Medicine, University of Augsburg, Augsburg, Germany; 4 Terrestrial Ecology and Climate Change, Department of Ecology, School of Biology, Faculty of Sciences, Aristotle University of Thessaloniki, 54124 Thessaloniki, Greece

**Keywords:** Plant ecology, Elevation, Mountain-valley gradient, Thermal factors, Reproduction, Air temperature

## Abstract

High-altitude environments are highly susceptible to the effects of climate change. Thus, it is crucial to examine and understand the behaviour of specific plant traits along altitudinal gradients, which offer a real-life laboratory for analysing future impacts of climate change. The available information on how pollen production varies at different altitudes in mountainous areas is limited. In this study, we investigated pollen production of 17 birch (*Betula pubescens* Ehrh.) individuals along an altitudinal gradient in the European Alps. We sampled catkins at nine locations in the years 2020–2021 and monitored air temperatures. We investigated how birch pollen, flowers and inflorescences are produced in relation to thermal factors at various elevations. We found that mean pollen production of *Betula pubescens* Ehrh. varied between 0.4 and 8.3 million pollen grains per catkin. We did not observe any significant relationships between the studied reproductive metrics and altitude. However, minimum temperature of the previous summer was found to be significantly correlated to pollen (*r*_*s*_ = 0.504, *p* = 0.039), flower (*r*_*s*_ = 0.613, *p* = 0.009) and catkin (*r*_*s*_ = 0.642, *p* = 0.005) production per volume unit of crown. Therefore, we suggest that temperature variability even at such small scales is very important for studying the response related to pollen production.

## Introduction

Plant traits such as phenology and tree growth have been repeatedly reported to be very sensitive to ongoing climate change (Dobbertin 2005; Menzel et al. 2006, 2020). For plant species, this sensitivity is amplified at the limits of species distribution (Mellert et al. 2016), where the ecological conditions do not meet the optimal requirements for plant survival and growth. Cold-adapted plant species growing at higher elevations in the European Alps are especially susceptible to the effects of climate change (Albrich et al. 2020; Engler et al. 2011), and tree species at the tree line were reported to be more sensitive to environmental changes (Wielgolaski et al. 2017). In general, mountain ecosystems allow studying climate change impacts as they cover a variety of changes related to abiotic and biotic factors along the elevational gradient (Tito et al. 2020). For instance, air temperature decreases by on average 0.5 °C for every 100 m of elevation, as reported for the Bavarian Alps, Germany (Kirchner et al. 2013). Studying plant responses using such lapse rates can be easily translated into thermal responses as often applied in phenological research (Cerlini et al. 2022; Damialis et al. 2020; Jochner et al. 2012). The plants’ behavior along an altitudinal gradient provides indications on potential impacts of climate change at small horizontal distances (Damialis et al. 2011; Jochner et al. 2012).

It is already widely documented that the flowering time of many spring flowering species has shifted earlier in the year due to increases in temperature (Khanduri et al. 2008; Menzel et al. 2020; Ziello et al. 2009). In addition to this well-known response in phenology, pollen production of different species was found to be affected by factors related to global change (Ladeau and Clark 2006; Ziska et al. 2003). However, most of these studies refer to warming experiments, and only very few studies assessed climate change impacts in real-life ecosystems, either in urban or rural and mountainous environments (e.g. Charalampopoulos et al. 2013; Damialis et al. 2011, 2020; Jochner et al. 2012).

Knowledge of changes in pollen production is important for *inter alia* predicting crop yield in agriculture (González-Fernández et al. 2020) and seed production in forestry (Allison 1990). Furthermore, exposure to airborne pollen of certain plant taxa provoke immune responses and allergic symptoms in sensitized individuals (Buters et al. 2012; D’Amato et al. 2007; Damialis et al. 2019). Even though there are sophisticated European models of airborne pollen abundance and timing (e.g. SILAM, Sofiev et al. 2015) including inter-seasonal variations of the potential amount of pollen emission (Verstraeten et al. 2019), most of them have not incorporated information on pollen production, which undoubtedly plays a vital role in forecasting the intensity of the airborne pollen season and associated allergic symptoms. Information on individual-specific values of pollen production can help understand the involved processes that contribute to modifications of pollen concentrations and hence might be important for implementation in pollen forecasting systems.

The assessment of pollen production and the extraction methods are not standardized, and most previous studies were descriptive in nature reporting quantitative estimates of single species and/or single locations (Fernández-González et al. 2020; Hidalgo et al. 1999; Khanduri and Sharma 2009; Molina et al. 1996; Subba Reddi and Reddi 1986). The spatial extent is larger (horizontally or vertically) when examining the influence of urbanization or altitude as these studies are based on environmental gradients (Damialis et al. 2011; Fotiou et al. 2011; Jochner et al. 2011; Ziska et al. 2003). However, there is still limited research on flower and/or pollen production along elevation gradients. This understanding is important since it would give information on the plant’s plasticity and how different environmental conditions impact reproductive traits (Charalampopoulos et al. 2013). Few studies assessed and attempted to explain pollen production of several woody species along elevation gradients, namely *Corylus avellana*, *Cupressus sempervirens*, *Olea europaea*, *Pinus halepensis*, *Platanus orientalis* and *Quercus coccifera*, mostly in Mediterranean regions (Aguilera and Valenzuela 2012; Charalampopoulos et al. 2013; Damialis et al. 2011; Rojo et al. 2015), and *Alnus incana* in the Nordic region (Moe 1998). Reproduction studies conducted along altitudinal gradients mainly focus on characteristics of seeds, e.g. seed quality, germination rate or weight (Allen et al. 2012; 2014). For birch species, Holm (1994) studied the reproductive patterns along an altitudinal gradient in Northern Sweden. So far and to the best of our knowledge, no previous study has investigated the variation of pollen production of birch at different altitudes. There has been a general lack of studies examining the pollen production of anemophilous species within alpine ecosystems as well as in the European Alps. In contrast, there are some studies on differences in birch pollen concentration in ambient air along altitudinal gradients in the Alps (Gehrig and Peeters 2000; Jochner et al. 2012; Wörl et al. 2022) and on pollen abundance and its correlation with allergic symptoms and immune reactions in sensitised patients (Damialis et al. 2019).

Birch (*Betula* spp.) trees are widely distributed across the Northern Hemisphere (Atkinson 1992), and their pollen are highly allergenic (D’Amato et al. 2017) and present a major cause of allergic rhinitis in central and northern Europe (Biedermann et al. 2019). They often grow in lowlands, although they are also present at higher altitudes (Emberlin et al. 2002). In Germany, birch is found up to an altitude of approx. 1800 m a.s.l. (DWD 1991). The latest citizen-science generated data demonstrated that *Betula pubescens* (downy birch) can occur at altitudes as high as 1840 m a.s.l., and *Betula pendula* (silver birch) was found at a maximum altitude of 1610 m a.s.l. in the Bavarian Alps (BAYSICS Webportal). Based on future projections using IPCC scenarios, birch trees in Bavaria are anticipated to become less common at lower elevations but shift their treeline and become more dominant at higher elevations in the Bavarian Alps over the next half century (Rojo et al. 2021). Spatiotemporal studies on birch pollen concentrations in the Bavarian Alps have also shown the effect of differing meteorological conditions such as wind patterns on birch pollen concentration (Jochner et al. 2012). Therefore, the estimation of actual and prospective pollen production and knowledge on spatial and temporal variations are important for forecasting future effects on respiratory allergies.

In the current work, we studied pollen production of *Betula pubescens* along a valley-mountain gradient in the Bavarian Alps for 2 consecutive years (2020 and 2021). The main aim of this work was to quantify the production of birch pollen, flowers and inflorescences (i.e. catkins) at sites ranging from 700 to 1220 m a.s.l. In addition, the relationship between reproductive metrics and thermal parameters was studied.

## Materials and methods

### Study area

**Fig. 1:**
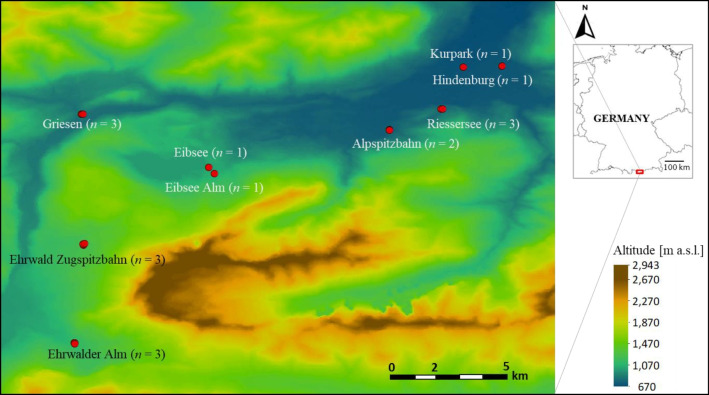
Location of the study sites in Germany/Austria (Eurostat GISCO) and in the Zugspitze region (NASA JPL 2020). Red dots: nine birch tree locations (with in total 17 birch individuals). White font locations are in Germany and black font locations are in Austria

The study area was located in southern Bavaria (Germany) and Tyrol (Austria) in the topographically complex region of the Zugspitze area (Fig. 1). With 2962 m a.s.l., the Zugspitze, which belongs to the Northern Limestone Alps in the Wetterstein Mountains, presents the highest mountain in Germany (Jochner et al. 2012). The birch trees were located in the city of Garmisch-Partenkirchen (700 m a.s.l.) and followed an altitudinal gradient up to the lake Eibsee (1000 m a.s.l.) and Ehrwald in Austria (1100 m a.s.l.). The highest location was at 1220 m a.s.l. (Ehrwald Cable Car Station); thus, the study covers an elevational gradient of 522 m. At lower sites, meadows are dominating; at higher elevations until approx. 1800 m, forests with spruce as the dominating tree species.

**Fig. 2:**
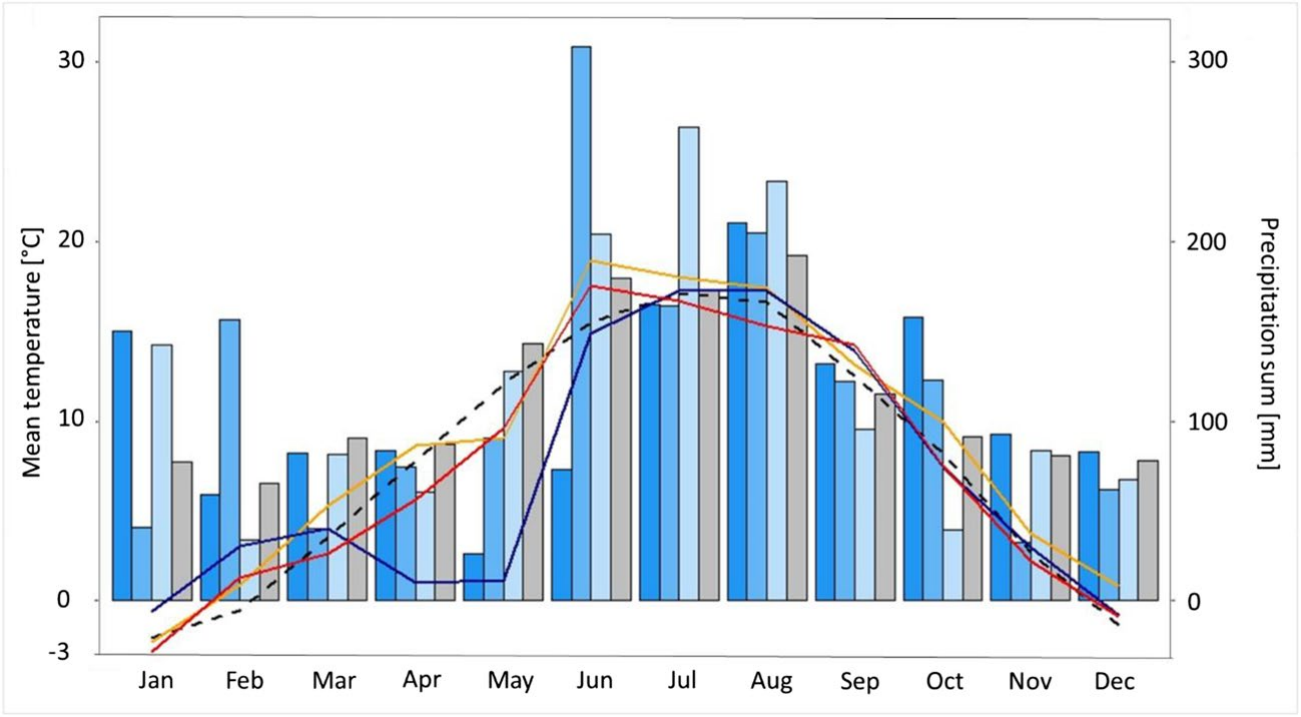
Monthly meteorological data recorded at DWD station Garmisch-Partenkirchen for the years 2019–2021. Solid lines show monthly average temperature: 2019 (orange), 2020 (dark blue) and 2021 (red) and blue bars the monthly precipitation sum: 2019 (dark blue), 2020 (blue) and 2021 (light blue). *x*-axis: months, left *y*-axis: monthly mean temperature in °C, right *y*-axis: monthly precipitation sum in millimetres. Mean values (1991–2020) are displayed as black dashed line (temperature) and grey bars (precipitation sum). Data: DWD 2022

The average annual temperature recorded at Garmisch-Partenkirchen is 7.7 °C and the average precipitation sum amounts to 1373 mm (1991–2020). For the years 2019–2021, the average temperature and total precipitation at Garmisch-Partenkirchen are 7.9 °C and 1315 mm (in 2019), 6.1 °C and 1419 mm (in 2020), and 7.4 °C and 1434 mm (in 2021), respectively (Fig. 2) (DWD 2022).

### Birch tree selection and inflorescence sampling

For assessing pollen production, we studied the species *Betula pubescens* Ehrh. The selection of individual trees was based on their presence, and the criteria of accessibility of the site and the reachability of catkins. Consideration was given to have at least one site for every 100 m of difference in elevation and to have representatives especially at the lowest (700–900 m; nine individuals) and highest sites (> 1100 m; six individuals) (Table 1). We collected inflorescence samples from 17 trees at nine locations (Table 1) in spring 2020 and 2021.

**Table 1:** Description of the location of the trees selected for studying their pollen production, including their coordinates, mean altitude (m a.s.l.) and number of the trees at each site in the Garmisch-Partenkirchen area

Location	Coordinates	Altitude (m a.s.l.)	*n* trees
Kurpark	N 47°29′46″E 11°05′26″	696	1
Hindenburg	N 47°29′47″E 11°06′19″	706	1
Alpspitzbahn	N 47°28′18″E 11°03′40″	749	2
Riessersee	N 47°28′47″ E 11°04′53″	781	2
Griesen	N 47°28′40″ E 10°56′27″	824	3
Eibsee	N 47°27′38″ E 10°59′14″	982	1
Eibsee Alm	N 47°27′16″ E 10° 59′34″	1,011	1
Ehrwalder Alm	N 47°23′17″ E 10°56′17″	1,102	3
Ehrwald Zugspitzbahn	N 47°25′36″ E 10°56′30″	1,218	3

After catkin elongation was initiated and before anthesis, male catkins with mature and closed anthers were collected in March and April 2020 and 2021. Catkins were harvested in all four cardinal directions from various branches at reachable heights (1.5 to 2 m a.g.l.). We also assessed tree parameters that were used for the extrapolation of pollen production from catkins to the crown volume: the height of the crown using Suunto PM-5/1520PC Height Meter and the crown diameter, which was computed by averaging the crown’s two widest perpendicular diameters. We counted the number of catkins inside a sampling cuboid in the crown with a volume of 50 cm × 50 cm × 50 cm and selected those areas of the tree crown that represent the typical distribution of catkins (Damialis et al. 2011).

### Pollen extraction method

We adapted the method of Damialis et al. (2011) for the extraction of birch pollen grains from closed inflorescences. The length and width (at the broadest point) of one medium-sized inflorescence from each cardinal direction and per tree were measured, and the number of flowers was counted. Then, each catkin was immersed in a 10% KOH solution overnight (Faegri et al. 1989; Moore et al. 1991; Ranpal et al. 2022). After boiling the solution the next day at 120 °C for 10 min, the soft catkin was mashed with a glass rod to discharge pollen. We added a bipolar solvent, glycerol (70%), to a volume of 20 mL (Ranpal et al. 2022) to prevent pollen from clustering (Shivanna and Rangaswamy 1992); safranin was applied as a stain. Using a VITLAB® micropipette, two aliquot samples (10 µL each) of each suspension were taken while the mixture was continuously stirred to achieve homogeneity. The extraction was then placed on microscope slides and covered with slips. We then counted the pollen grains on these slides using a 100 × magnification (Zeiss AXIO Lab.A1, Germany). In case of a substantial difference in the pollen counts between these two slides (> 30%), the progress was repeated to increase homogeneity of the suspension.

We estimated pollen production for different scales following the formulae mentioned by Damialis et al. (2011). The number of pollen grains per catkin (*P*_*ca*_) was calculated by multiplying the number of pollen grains on a microscope slide with the ratio of the volumes of the suspension (20 mL) and the sample taken (10 µL). Following, the number of pollen grains per flower (${P}_{\mathrm{fl}}$) was derived as a quotient of *P*_*ca*_ divided by the numbers of flowers per catkin (*Fl*_*ca*_). The number of pollen grains per volume unit (1 m^3^) of crown (*P*_*vuc*_) was determined by multiplying *P*_*ca*_ with the ratio of the number of catkins per crown sampling unit (*C*_*su*_) and the volume of the sampling unit (0.125 m^3^). In addition, the number of flowers (*Fl*_*vuc*_) and catkins (*C*_*vuc*_) per volume unit of crown was extrapolated.

### Environmental data

To assess the influence of temperature on pollen production, we positioned nine loggers with radiation shields (HOBO Pro v2 U23-001, Onset, Bourne, MA, USA). Each logger was set up at a height of 2 m a.g.l. on the northern side of one birch tree from one location, which recorded temperature at 10-min intervals from February 2020 until June 2021. A new HOBO logger was installed at the location Griesen in January 2021 as the previous one was lost, and no data were available for this site until December 2020. The location’s missing daily temperature data were interpolated applying linear regression with daily temperatures and altitudes of the other eight loggers. The root-mean-square errors (RMSE) between predicted and observed daily mean temperature during January until June 2021 were 1.2. The software package HOBOware (Version 3.7.23; Onset, Bourne, Massachusetts, USA) was used to download the data from the loggers and to export the raw data as text files.

We focussed on temperatures measured in the summer previous of flowering, since this period is assumed to be important for pollen production (Ranpal et al. 2022), as catkins already start to develop and elongate (Dahl and Strandhede 1996). Thus, we were able to compare temperature data of 2020 with pollen production of 2021. For the first study year, we cannot resort to 2019 data; thus, we link pollen production of 2020 to March temperatures of 2020. For comparison, we also link 2021 pollen data to 2021 March temperatures.

Furthermore, we calculated growing degree-days (GDD; in °C) of summer 2020 (June–August) by cumulating positive differences between the daily mean temperature (derived as an average of *T*_*max*_ and *T*_*min*_) and a threshold temperature. In our study, we used a base temperature of 5 °C (Bucher et al. 2018; Estrella and Menzel 2006).

### Statistical analyses

All levels of flower, catkin and pollen production were checked for normality using Shapiro–Wilk test, which revealed that these reproductive measures were not normally distributed. Non-normality was dealt with by using non-parametric tests.

We examined differences between sampling years using the non-parametric Mann–Whitney *U* test and applied Spearman’s correlations to analyse association between altitude and reproductive metrics. In addition, the influence of the altitude on the tree-specific differences in reproductive metrics between 2021 and 2020 was checked. To investigate the effect of environmental factors on pollen production of birch along the elevational gradient, we compared reproductive metrics with temperature variables (*T*_*mean*_, *T*_*min*_, *T*_*max*_, GDD).

All statistical analyses were carried out with R version 4.2.2 (R Core Team 2020).

## Results

### Pollen, flowers and catkins production

Pollen production per catkin (*P*_*ca*_) for all selected 17 birch trees in the area of Garmisch-Partenkirchen was 5.23 ± 1.52 million pollen grains in 2020 and 2.51 ± 1.23 million pollen grains in 2021 (see Table 2). *P*_*ca*_ varied within a wide range from approx. 400,000 (minimum of 2021) to 8.3 million pollen grains (maximum of 2020). *P*_*ca*_ in 2021 was 52% lower compared to 2020 when regarding mean values. The number of catkins in a crown sampling unit (*C*_*su*_; 0.125 m^3^) ranged between 1 (minimum of 2021) and 50 (maximum of 2020) with an average of 28 catkins in 2020 and 5 catkins in 2021 (− 82%). In addition, all other estimated parameters, i.e. pollen production per flower (*P*_*fl*_), per volume unit of crown (*P*_*vuc*_) and the number of flowers per catkin (*Fl*_*ca*_), were consistently higher in 2020, which does not only apply to mean, but also to minimum and maximum values (Table 2).

**Table 2:** Descriptive statistics of pollen production per flower (*P*_*fl*_), per catkin (*P*_*ca*_) and per volume unit of crown (*P*_*vuc*_); flower production per catkin (*F*_*ca*_) and per volume unit of crown (*Fl*_*vuc*_) and catkin production per crown sampling unit (*C*_*su*_; 0.125 m^3^) and per volume unit of crown (*C*_*vuc*_) estimated for 17 selected birch trees along an altitudinal gradient in the Garmisch-Partenkirchen area during 2020–2021. The second last column indicates the results of the Mann–Whitney *U* test for comparison of means

Reproductive metric	Year	Minimum	Maximum	Mean	Median	Standard deviation	W statistic (*p* value)	Difference 2020 to 2021 (in %)
Pollen production								
*P*_*fl*_	2020	20,442	79,007	45,738	44,886	13,041	256 (≤ 0.001)	48%
	2021	4,445	56,139	23,639	23,154	12,798		
*P*_*ca*_	2020	2,197,500	8,257,667	5,228,025	5,255,750	1,521,924	262 (≤ 0.001)	52%
	2021	398,667	5,409,250	2,507,427	2,412,500	1,230,822		
*P*_*vuc*_	2020	187,620,000	2,039,200,000	1,095,620,550	929,480,000	562,490,982	287 (≤ 0.001)	91%
	2021	6,378,672	482,500,000	96,997,804	69,294,000	109,138,708		
Flower production								
*Fl*_*ca*_	2020	84	146	115	114	16	181 (0.214)	6%
	2021	77	125	108	112	14		
*Fl*_*vuc*_	2020	4560	42,560	25,163	24,240	12,085	278 (≤ 0.001)	82%
	2021	896	20,800	4314	2712	4800		
Catkin production								
*C*_*su*_	2020	5	50	28	25	14	276 (≤ 0.001)	82%
	2021	1	25	5	3	6		
*C*_*vuc*_	2020	40	400	223	200	108	276 (≤ 0.001)	82%
	2021	8	200	41	24	46		

### Year-to-year variation in reproductive metrics

**Fig. 3:**
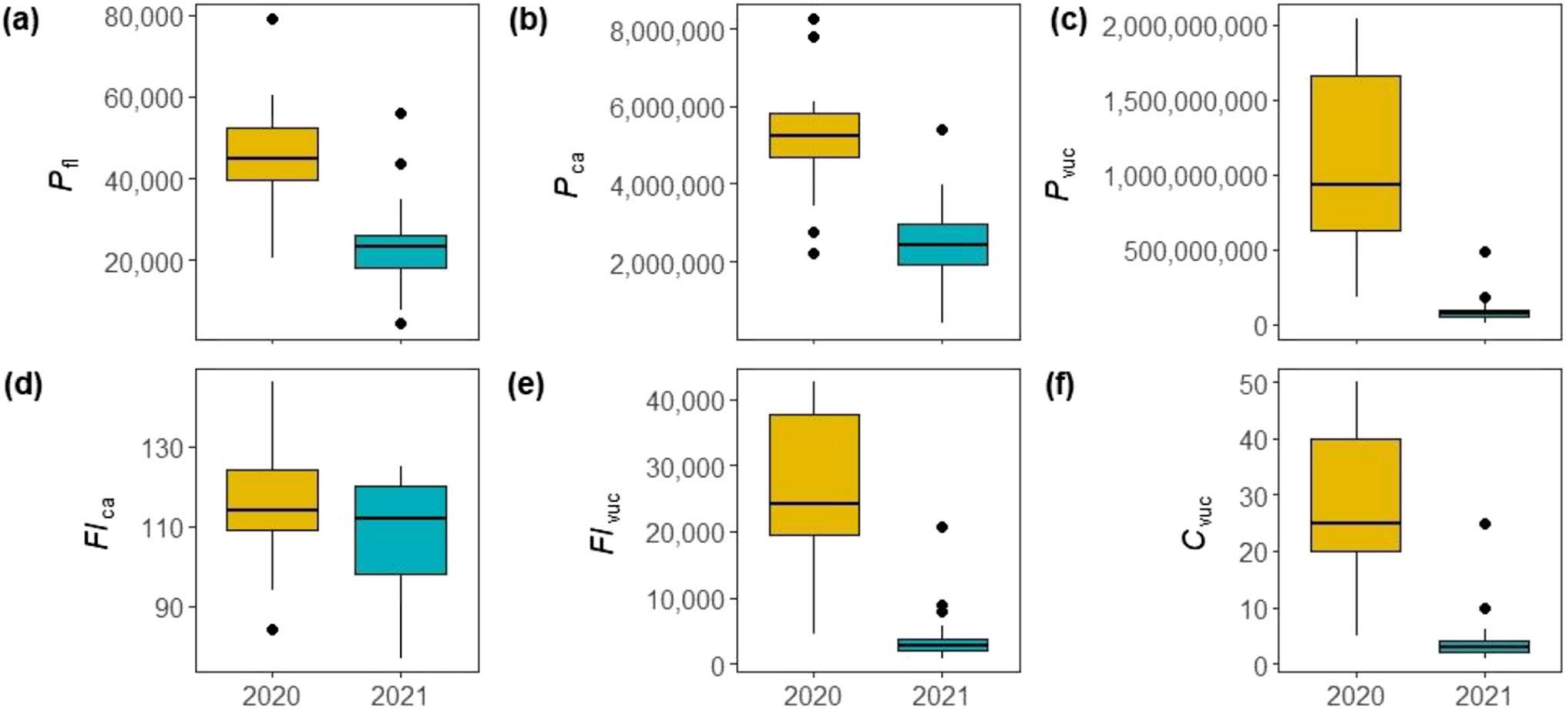
Boxplots based on (**a)** *P*_*fl*_, **(b)** *P*_*ca*_, **(c)** *P*_*vuc*_, (**d)** *Fl*_*ca*_, **(e)** *Fl*_*vuc*_ and **(f**) *C*_*vuc*_ estimated for 17 trees along an altitudinal gradient in the Garmisch-Partenkirchen area for 2020 and 2021. The interquartile range (IQR) is represented by the height of the boxes, maximum and minimum values by the upper and lower whiskers, the median by bold horizontal lines in the boxes, dots represent observations exceeding or falling below 1.5 times the IQR

The Mann–Whitney *U* test revealed that the means of all reproductive metrics except for flowers per catkin (*Fl*_*ca*_) (*p* = 0.214) were significantly different between 2020 and 2021. In each case, the percentage change was positive, i.e. the highest values were measured in 2020. Figure 3 shows exemplary the differences of *P*_*fl*_, *P*_*ca*_, *P*_*vuc*_, *Fl*_*ca*_, *Fl*_*vuc*_ and *C*_*vuc*_ between 2020 and 2021.

### Effects of altitude and temperature on reproductive metrics

#### Altitude

In 2020, there were no significant correlations between the reproductive metrics and altitude (Table 3). In 2021, some correlation coefficients increased in magnitude, but were not statistically significant (marginally significant for *Fl*_*vuc*_, *r*_*s*_ =  − 0.446, *p* = 0.073 and *C*_*vuc*_, *r*_*s*_ =  − 0.443, *p* = 0.075).

**Table 3:** Spearman’s correlations between altitudes, temperature metrics, and reproductive metrics of 17 birch trees located in the Garmisch-Partenkirchen area in 2020 and 2021. *r*_*s*_ Spearman’s correlation coefficient, *p* significance value

2020		Altitude	*T*_*min*_ March 2020	*T*_*mean*_ March 2020	*T*_*max*_ March 2020	GDD March 2020				
*P*_*fl*_	*r*_*s*_	− 0.071	0.093	− 0.006	− 0.014	− 0.009				
	*p*	0.786	0.723	0.981	0.959	0.974				
*P*_*ca*_	*r*_*s*_	− 0.148	0.190	0.087	0.059	0.020				
	*p*	0.570	0.464	0.741	0.821	0.940				
*P*_*vuc*_	*r*_*s*_	− 0.221	0.085	0.117	0.068	0.209				
	*p*	0.395	0.745	0.654	0.795	0.421				
*Fl*_*ca*_	*r*_*s*_	0.122	− 0.170	− 0.138	− 0.157	− 0.185				
	*p*	0.642	0.515	0.599	0.546	0.478				
*Fl*_*vuc*_	*r*_*s*_	− 0.104	− 0.049	0.030	− 0.020	0.022				
	*p*	0.691	0.851	0.910	0.940	0.932				
*C*_*vuc*_	*r*_*s*_	− 0.165	0.006	0.078	0.031	0.068				
	*p*	0.527	0.981	0.765	0.906	0.794				
**2021**		**Altitude**	***T***_***min***_** March 2021**	***T***_***mean***_** March 2021**	***T***_***max***_** March 2021**	**GDD March 2021**	***T***_***min***_** Summer 2020**	***T***_***mean***_** Summer 2020**	***T***_***max***_** Summer 2020**	**GDD Summer 2020**
*P*_*fl*_	*r*_*s*_	0.405	− 0.147	− 0.234	− 0.189	0.115	− 0.288	− 0.399	− 0.328	− 0.330
	*p*	0.107	0.573	0.367	0.467	0.660	0.262	0.112	0.199	0.196
*P*_*ca*_	*r*_*s*_	0.363	− 0.119	− 0.220	− 0.171	0.121	− 0.279	− 0.354	− 0.289	− 0.289
	*p*	0.152	0.650	0.396	0.513	0.643	0.278	0.164	0.260	0.260
*P*_*vuc*_	*r*_*s*_	− 0.303	0.361	**0.512**	*0.455*	**0.774**	**0.504**	0.232	− 0.010	0.272
	*p*	0.237	0.155	0.036	0.067	0.000	0.039	0.369	0.970	0.291
*Fl*_*ca*_	*r*_*s*_	− 0.032	0.170	0.133	0.100	0.159	− 0.004	0.101	0.224	0.153
	*p*	0.903	0.513	0.610	0.703	0.541	0.987	0.700	0.388	0.557
*Fl*_*vuc*_	*r*_*s*_	* − 0.446*	*0.470*	**0.635**	**0.509**	**0.650**	**0.613**	0.391	0.173	*0.425*
	*p*	0.072	0.057	0.006	0.037	0.005	0.009	0.121	0.507	0.089
*C*_*vuc*_	*r*_*s*_	* − 0.443*	*Not shown since catkin development is prior to March 2021*				**0.642**	0.400	0.145	*0.425*
	*p*	0.075					0.005	0.112	0.578	0.089

Significant at the 0.05 level (bold values) and marginally significant (at the 0.1 level; italics values)

**Fig. 4:**
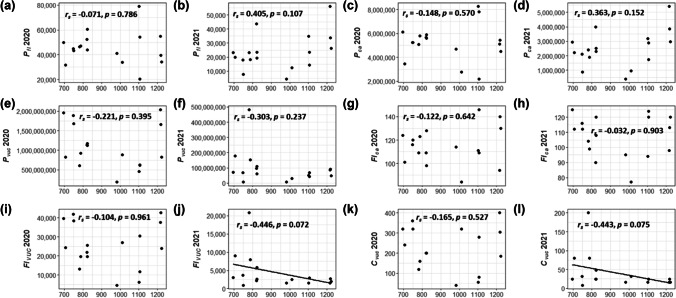
Scatterplots of altitude and *P*_*fl*_ in 2020 (**a**), 2021 (**b**); *P*_*ca*_ in 2020 (**c**), 2021 (**d**); *P*_*vuc*_ in 2020 (**e**), 2021 (**f**); *Fl*_*ca*_ in 2020 (**g**), 2021 (**h**); *Fl*_*vuc*_ in 2020 (**i**), 2021 (**j**); and *C*_*vuc*_ in 2020 (**k**), 2021 (**l**) estimated for 17 trees in the Garmisch-Partenkirchen area. Regression lines were added in case of marginally significant relationships

Figure 4 illustrates the relationship between altitude and reproductive metrics estimated in 2020 and 2021. Especially in 2020, but also in 2021, there is a large scattering, which was also reflected by the non-significant relationship (Table 3). Regression lines were added in case of marginally significant relationships.

**Fig. 5:**
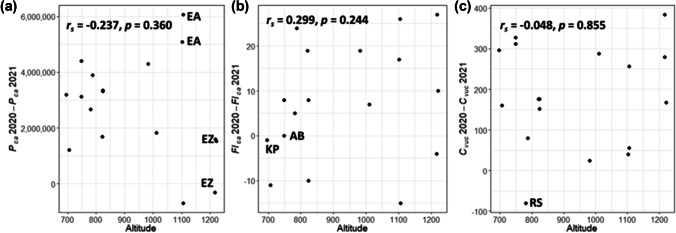
Scatterplots of the difference in the selected reproductive metrics (**a)** *P*_*ca*_, **(b)** *Fl*_*ca*_ and (**c)** *C*_*vuc*_ between 2020 and 2021 estimated for 17 trees in the Garmisch-Partenkirchen area (locations: AB, Alpspitzbahn; EA, Ehrwalder Alm; EZ, Ehrwald Zugspitzbahn; KP, Kurpark; and RS, Riessersee) and respective altitudes

Figure 5 demonstrates the differences in the selected reproductive metrics (*P*_*ca*_, *Fl*_*ca*_ and *C*_*vuc*_) between 2020 and 2021. Though the correlations are all non-significant, some interesting patterns can be revealed: Only two trees, located above 1100 m a.s.l., were linked to a negative *P*_*ca*_ value, i.e. higher pollen production, in 2021 (Fig. 5a). Three trees at the highest location (Ehrwald Zugspitzbahn; EZ) showed relatively small differences, but two trees at Ehrwalder Alm (EA) the largest differences. No or a very small differences in *Fl*_*ca*_ between 2020 and 2021 were obtained for trees at Alpspitzbahn (AB; 749 m a.s.l.) and Kurpark (KP; 696 m a.s.l.). Four trees were linked to less flowers in 2021, the rest to more flowers. A clearer pattern was seen for the differences in *C*_*vuc*_ between 2020 and 2021. Here, only one tree (located at Riessersee; RS) was associated to a lower number of catkins in 2020. However, correlation analyses revealed no significant relation to altitude.

### Temperature

In general, mean summer temperatures (June–August 2020) recorded at each site were negatively and strongly correlated with altitude (*r*_*s*_ =  − 0.940, *p* < 0.001). GDD of the same period was also negatively and strongly correlated with altitude (*r*_*s*_ =  − 0.880, *p* < 0.001). The highest temperature mean (17.8 °C), considered for the period of June–August 2020, was measured at Hindenburg (706 m a.s.l.), which is one of the lowest locations of the study. The lowest mean annual temperature (14.8 °C) was recorded at the highest site, at Ehrwald Zugspitzbahn (1218 m a.s.l.) (Table 1). The sites with the highest (Hindenburg; 5.0 °C) and the lowest (Ehrwald Zugspitzbahn; 1.7 °C) temperature mean recorded in March 2020 were the same as mentioned above.

The relationships with reproduction metrics and temperature variables (minimum, mean, maximum temperature and GDD) were not statistically significant in 2020, the year in which the highest pollen and catkin production was observed. For 2021, however, we found some significant correlations. *C*_*vuc*_ was significantly (*p* = 0.005) correlated to summer *T*_*min*_ (*r*_*s*_ = 0.642). Since the amount of catkins in a sampling volume also influences the reproduction measures *Fl*_*vuc*_ and *P*_*vuc*_, positive and significant relationships were also derived in these cases: *Fl*_*vuc*_ and summer *T*_*min*_ (*r*_*s*_ = 0.613, *p* = 0.009), *P*_*vuc*_ and summer *T*_*min*_ (*r*_*s*_ = 0.504, *p* = 0.039). Interestingly, other temperature variables calculated for the period June to August (mean and maximum temperatures, GDD) were not significantly associated to any of the reproductive metrics. Instead, March temperatures were sometimes superior in describing the relationship. The highest correlation was achieved with *P*_*vuc*_ and GDD (*r*_*s*_ = 0.774, *p* = 0.000). In summary, warmer conditions were related to higher pollen and flower production, which was only obvious for higher levels, i.e. for the volume unit of the crown, as a result of the temperature dependency of catkin numbers in 2021.

**Fig. 6:**
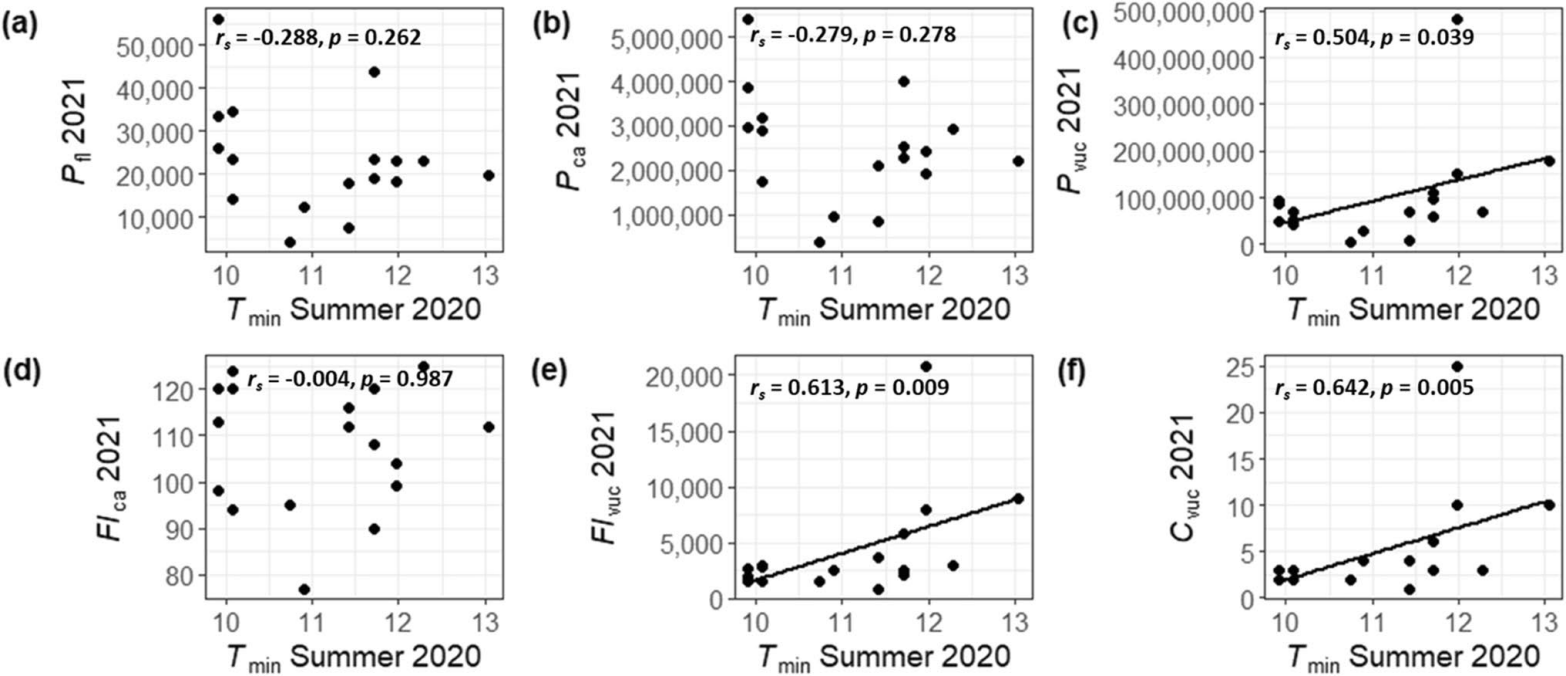
Relationship between (**a)** *P*_*fl*_ 2021, (**b)** *P*_*ca*_ 2021, **(c)** *P*_*vuc*_ 2021, **(d)** *Fl*_*ca*_ 2021, **(e)** *Fl*_*vuc*_ 2021 and **(f)** *C*_*vuc*_ 2021, and summer *T*_*min*_ of 2020 for 17 birches along the altitudinal gradient in the Garmisch-Partenkirchen area. Regression lines were added in case of significant relationships

For visualization (Fig. 6), we focused on the relationships with reproductive metrics estimated in 2021 and minimum summer temperature in 2020. The subplots (a), (b) and (d) in Fig. 6 demonstrate that pollen and flower production of male inflorescences in 2021 was random with respect to the *T*_*min*_ of the previous summer. However, an increase in the number of pollen, flowers and catkins per volume unit of crown was observed with higher values of *T*_*min*_ in summer 2020 (Fig. 6c, e, f).

## Discussion

### The value of gradient studies in pollen research

Long-term studies can profit from almost constant site conditions (soil type, edaphic regime) and varying meteorological conditions across years that may allow for calculating trends or response rates based on the same individual and therefore excluding genetic variability (Jochner et al. 2013a). Studies along altitudinal gradients can be affected by several factors such as complex environmental heterogeneity and extreme geography (Körner 2007). As the space-for-time approach also includes a multitude of different individuals at various sites, differences in local environmental factors, e.g. soil conditions, nutrient availability and water supply as well as differences in pollution or other factors related to local climate and genetics might exert additional influences. Thus, gradient studies do not only account for differences in temperature, which is, however, the most important variable when the effect of climate change is aimed to be assessed.

### Pollen, flowers and catkins production

In the present study, we investigated how reproductive metrics of *Betula pubescens* trees differ in 2 consecutive years along an altitudinal gradient in the Bavarian Alps. We estimated mean pollen production values at the level of catkins ranging between 5 and 2.5 million. This estimate is comparable to that published by Erdtman (1954), who reported a value of 6 million pollen grains per inflorescence for *B. pubescens*. Our mean value, however, is an average of the estimates from 17 downy birch trees growing in various elevations for 2 years (*n* = 34). According to Ranpal et al. (2022), a catkin from *Betula pendula* (silver birch) produces on average 1.7 million pollen grains. Such estimations of pollen production might be important to assist pollen emission parameterization since commonly, their proxies were only related to plant characteristics such as leaf area index and canopy height (Helbig et al. 2004).

Estimation of pollen production by counting all pollen on microscope slides is a labour- and time-intensive method. Improved pollen counting methods, such as a cell counter (Kakui et al. 2020) or automatic identification of the number of pollen grains on microscopic slides (Kadaikar et al. 2019), would make the method more efficient.

### Year-to-year variation in reproductive metrics

The year 2020 was found to be a pollen- and catkin-rich year: We estimated on average 109% more pollen grains per catkin than in the following year. In addition, the number of catkins per volume unit of crown was 460% higher in 2020, but the number of flowers per catkin was only changed slightly (+ 6%) and associated to a non-significant difference.

Flower numbers were not substantially different between years. The number of anthers per flower is genetically fixed and does not vary substantially (Fernández-González et al. 2020; Hidalgo et al. 1999; Subba Reddi and Reddi 1986). In the case of birch, flowers per catkin seem to have the most homogenous value among clones and years (Ranpal et al. 2022). However, the number of flowers per volume unit of crown was also calculated by multiplying the number of flowers of single catkins by the abundance of catkins within the volume. Thus, the number of catkins, which can largely differ between trees and years, is the most decisive factor for the value obtained for flowers per volume unit of a crown.

In general, reproductive metrics in birch trees can vary greatly from year to year, as found by Jato et al. (2007), Damialis et al. (2011) and Ranpal et al. (2022). Alternating patterns of flower (and seed) production are related to masting behaviour, an inherent common feature in temperate tree species that occurs, in the case of birch, every second or third year (Detandt and Nolard 2000; Latałowa et al. 2002). Given that the catkin (more than 10 times) and pollen production (3 times) were extraordinarily high in 2020, one may assume that this year was a masting year. At a seed plantation in Baden-Württemberg, Germany (distance to Garmisch-Partenkirchen approx. 210 km), Ranpal et al. (2022) also found that mean *C*_*su*_ of a total of 28 trees in 2020 was two times higher than in the preceding year and the subsequent year. Data obtained from our pollen monitoring site in Eichstätt, Bavaria (distance to Garmisch-Partenkirchen approx. 160 km), also indicated that 2020 was linked to a high pollen load in the air: Here, an APIn (annual pollen integral) of 8720 pollen grains*day/m^3^ was measured, compared to only 1923 pollen grains*day/m^3^ in the following year (unpublished data). In general, for defining mast years, a longer time-series would be needed for a detailed identification and evaluation (LaMontagne and Boutin 2009). The delineation of mast years is mostly based on concepts that include the coefficient of variation that accounts for the mean and standard deviation, but consistent and generally applicable methods are not available (LaMontagne and Boutin 2009). In the case of pollen, one reason might be the underrepresentation of studies addressing flower masting (Pearse et al. 2016; Satake and Iwasa 2002).

Thus, the lack of studies related to flower masting along altitudinal gradients is not surprising. This is in contrast to seed masting, where changes of temporal patterns of masting were *inter alia* already linked to the variation in climatic conditions along elevational gradients (Masaki et al. 2020). The authors found that mean fruiting density and fruiting frequency of *Quercus crispula* decreased with elevation, while the annual variation in fruiting density increased. Therefore, harsh environmental conditions (e.g. low temperatures) at high elevations might be linked to a reduced photosynthetic production and increased masting (Masaki et al. 2020). In our study, which was only based on two consecutive years, we found no significant dependency between the deviation from 2020 and 2021 in pollen, flower or catkin production and altitude (Fig. 5). In 2020, the vast majority of selected trees synchronously produced a higher amount of pollen, flowers and catkins. However, it was obvious that two trees located at high elevations were the only exceptions showing a negative deviation (i.e. higher flower and pollen production per catkins in 2021). The number of catkins produced in 2021 for those trees, however, was very low as well; thus, the pollen or flower production based on larger units (i.e. volume unit of the crown) was still higher in 2020. These findings also point to the need for defining and categorizing (flower) masting since reproductive metrics can be altered differently. For this reason, a larger dataset including more observation years and more birch trees, e.g. located at even harsher sites, would be desirable.

It should be noted (but must remain unevaluated) that the masting year has occurred (in 2020) after the year (2019) with the highest temperature (7.9 °C) and lowest precipitation sum (1315 mm), registered in Garmisch-Partenkirchen in the period of 2019–2021. This also calls for the installation of a long-term monitoring in order to be able to understand the influence of meteorology on masting years in more detail.

### Effects of altitude and temperature on reproductive metrics

#### Altitude

We found that there were no prominent changes in the analysed reproductive traits with increasing altitude (Table 3; Fig. 4). The results of very few prior studies studying pollen production along altitudinal gradients showed that there is no conclusive evidence on the associated relationships with increased elevation since a decrease in pollen production (Markgraf 1980; Moe 1998), an increase (Aguilera and Valenzuela 2012) or no significant change (Charalampopoulos et al. 2013; Hasegawa et al. 2022) was observed. Aguilera and Valenzuela (2012) argued that higher olive pollen production observed at elevated regions might be related to intrinsic mechanisms of these trees to compensate for a limited pollination efficiency and a shorter growing period. However, these results may also be affected by human interventions (cutting) that may have a masking effect on pollen production. However, in some of the studied species at Mount Olympos, pollen and inflorescence traits at the higher reproduction level (e.g. per individual tree) were decreased with increasing altitude (Charalampopoulos et al. 2013). In this study, we decided not to integrate the number of pollen, flowers or inflorescences per individual, since this measure is strongly dependent on the age and height of a tree that considerably varies along the gradient under investigation. In addition, extrapolating production estimates to the whole tree is based on the assumption of a simplified geometric shape of the tree (Molina et al. 1996). However, this potential geometric shape differs from its original form to a certain extent, implying uncertainties in the estimation for the level of an individual tree. All variables based on a specific volume are believed to be superior indicators of pollen production, since they account for the pollen produced per catkins and the number of catkins in a standard volume (1 m^3^). Some studies, such as those by Bogawski et al. (2019) and Katz et al. (2020), have used LiDAR data to determine crown parameters, which were used for estimating pollen production per tree or tree stand.

The result of this study indicated that the number of male inflorescences per crown sampling unit (*C*_*su*_) in 2021 decreased along the gradient (*r*_*s*_ =  − 0.443, *p* = 0.075, Table 3). Thus, compensation for pollen limitation might more strongly affect the pollen produced by single inflorescences. Fernández-González et al. (2020) found that smaller sized tree species of the genus *Quercus* attempt to produce a higher amount of pollen per anther to ensure fertilization.

#### Temperature

Although we found a strong and significant relation with temperature and altitude, those variables associated to temperature showed stronger and more significant correlations than altitude alone. This also points to the fact that temperature measurement should be implemented in any altitudinal gradient studies.

In 2021, we detected an increased catkin formation at warmer (lower) locations, which was also reflected in the reproductive metrics whose computations were based on the number of catkins. Our results indicated that minimum temperature was superior in any statistical analyses than mean and maximum temperatures or even GDD.

Non-significant relationships with temperature were found in 2020, the assumed mast year. The reason might be that the amount of pollen and inflorescences produced by the selected birch trees in our study was most probably regulated by the resource balance of the trees, and masting-associated parameters masked other influences and variability present in normal reproductive years. According to the resource budget model, masting can occur due to plants’ resource balance even in the absence of interannual environmental variations (Isagi et al. 1997). In general, pollen concentration and therefore pollen availability is reduced at higher elevations due to a decreasing prevalence of birch trees (Charalampopoulos et al. 2013; Jochner et al. 2012). A lower availability of birch pollen might also cause low seed production. Following the resource storage hypothesis, this may affect resource accumulation resulting in more flowering/fruiting (Bogdziewicz et al. 2020) as observed in our study in 2020. Therefore, the fact that birch is only seldom represented at higher altitudes in our study area, might also affect its resource budget, which could mask the influence of environmental factors such as temperature.

Existing studies indicate varying relationships between temperature and pollen production. Jochner et al. (2013b) found a significant reduction in pollen production per catkin in silver birch (*Betula pendula* Roth) at urban locations (under higher temperatures) in Munich (Germany). The authors argued that conditions in urban areas might have a negative effect on the physiology of birch and thus on pollen production. On the other hand, an urban gradient study indicated that an increase in temperature increased the pollen production of other species such as common ragweed (*Ambrosia artemisiifolia*, Ziska et al. 2003).

In general, microclimate is believed to have a strong impact on pollen production (Aguilera and Valenzuela 2012); therefore, a variability is quite expected and would be even more pronounced when studying a larger altitudinal gradient. With the calculated temperature lapse rates in this study (season-depended varying between 0.4 and 0.6 °C; not shown in the results section) and the given gradient of 522 m, a temperature difference of 2.1 and 3.1 °C might be too low to observe strong effects on pollen, flower and inflorescence production.

### Effects of other environmental parameters

In addition, other information than air temperature might be important: A study in alpine environments by Scherrer et al. (2011) found significant fluctuations in soil temperature of up to 4 °C depending on slope aspect and topography. Because of this, even trees at the same location and similar altitude are exposed to different microclimatic conditions that might affect reproductive traits.

Air pollutants such as nitrogen dioxide (NO_*2*_) might reduce (Jochner et al. 2013b) or increase pollen production of birch (Zhao et al. 2017). In addition, ozone (O_*3*_) was also found to affect birch reproduction (Darbah et al. 2008). Birch trees growing in areas with higher NO_*2*_ levels were found to be more often affected with birch idaeovirus (Gilles et al. 2023), and such biotic stress could further influence the reproduction of infected trees. These pollutants are likely to change with increasing elevation and should also be incorporated in further studies. In the present study, we only measured NO_*2*_ and O_*3*_ during a 1-week period in late spring 2020 and found significant correlations between NO_*2*_ and *P*_*vuc*_ 2020 (*r*_*s*_ = 0.520, *p* = 0.032) as well as between O_*3*_ and *P*_*ca*_ 2020 (*r*_*s*_ =  − 0.519, *p* = 0.033) and *P*_*fl*_ 2020 (*r*_*s*_ =  − 0.489, *p* = 0.047) (not shown). Since these results are only based on a short measurement duration, we decided not to incorporate these findings in the “Results” section but encourage further research to specifically focus on pollution as potential influential factor. Since the effects of pollution might also be species-specific, there is also a strong need to compare different plant species.

Moreover, other factors can have an influence on pollen production, such as artificial pruning/topping, since the induction of stress results in a higher reproductive output (Ranpal et al. 2022). In addition, site characteristics such as stand density and exposure (Faegri et al. 1989) and genetics (Ranpal et al. 2022) were found to be relevant in the discussion on pollen production.

Knowledge derived from seed masting studies suggest that nutrient availability which usually declines with elevation as a result of decreased organic matter decomposition and nutrient mineralization (Sundqvist et al. 2013) might also affect seed availability (Allen et al. 2014). Related to birch pollen production per catkin, it was found that iron concentration (assessed in birch leaves) was linked to a decrease (Jochner et al. 2013b), but other information on the influence on nutrients, specially assessed in the soil, is largely lacking.

Low temperature and high moisture availability 2 years before seed fall was linked to a higher amount of seed production (Richardson et al. 2005). Relationships with reproduction variables related to pollen based on lag effects, however, are not commonly evaluated in existing research and highlights the need for long-term studies.

In addition, more experimental studies may be best suited to disentangle the influence of temperature and other factors influencing reproduction traits of plants and their magnitude free from masked effects. Birch trees become sexually mature (and bear male catkins) from the age of approx. 10–15 years (Perala and Alm 1990). Therefore, in the case of birch, experimental setups remain challenging since their relocation to laboratory conditions cannot easily be materialized.

In summary, future research could benefit from the inclusion of more birch trees spanning an even larger altitudinal gradient and observation years. Ideally, a long-term monitoring, which is still not established, is desirable. Spatial information on air pollution along with meteorological measurements is helpful to conclude on their influences on pollen production. Less time-consuming methods of pollen quantification should be tested and more experimental research avoiding masked effects on pollen production is suggested.

## Conclusions

In conclusion, this study provides valuable insights into the production of birch pollen, flowers and inflorescences in relation to thermal parameters across an elevational gradient. The findings of this study indicate that no significant changes in the reproductive traits were detectable with increasing altitude alone. Moreover, likely due to the temperature dependency of catkin numbers in 2021, warmer sites were associated with higher pollen and flower production, which was only apparent for higher levels, i.e. for the volume unit of the crown. Temperatures further from the optimum of birch growth might be linked to more pronounced changes; thus, studying pollen production along even larger altitudinal gradients is highly relevant in future research.
